# Supplementary material for: Multi-omics evaluation of cell lines as models for metastatic prostate cancer
Source: Commun Biol. 2026 Mar 24;9:656. doi: 10.1038/s42003-026-09914-2 (PMC13171877; doi:10.1038/s42003-026-09914-2)
Supplement: Supplementary file 1 — Supplementary information [file 42003_2026_9914_MOESM1_ESM.pdf]

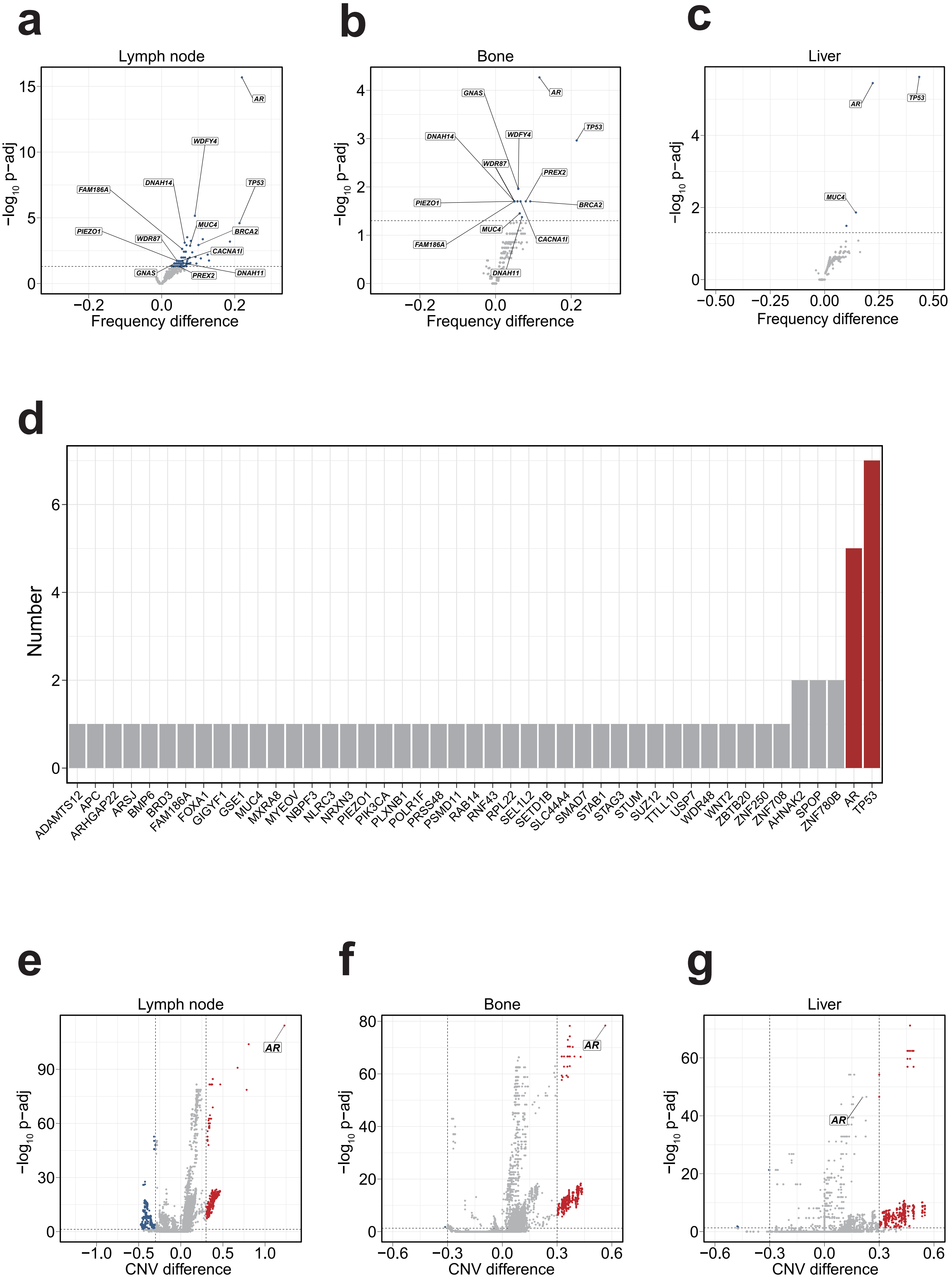
a
Lymph node
−log10 p−adj
Frequency difference
AR
WDFY4
DNAH14
TP53
FAM186A
MUC4
BRCA2
PIEZO1
WDR87
CACNA1I
GNAS
PREX2
DNAH11
b
Bone
GNAS
AR
TP53
DNAH14
WDFY4
WDR87
PREX2
PIEZO1
BRCA2
MUC4
CACNA1I
FAM186A
DNAH11
c
Liver
AR
TP53
MUC4
d
Number
ADAMTS12
APC
ARHGAP22
ARSJ
BMP6
BRD3
FAM186A
FOXA1
GIGYF1
GSE1
MUC4
MXRA8
MYEOV
NBPF3
NLRC3
NRXN3
PIEZO1
PIK3CA
PLXNB1
POLR1F
PRSS48
PSMD11
RAB14
RNF43
RPL22
SEL1L2
SETD1B
SLC44A4
SMAD7
STAB1
STAG3
STUM
SUZ12
TTLL10
USP7
WDR48
WNT2
ZBTB20
ZNF250
ZNF708
AHNAK2
SPOP
ZNF780B
AR
TP53
e
Lymph node
−log10 p−adj
CNV difference
AR
f
Bone
AR
g
Liver
AR

Supplementary Figure 1:

(**a-c**) Metastasis-site-specific comparisons of gene mutation frequencies between SU2C and TCGA samples. Each point represents a gene; the x-axis denotes the difference in mutation frequency (SU2C-TCGA), and the y-axis shows statistical significance. The horizontal dashed line indicates an adjusted *P*-value cutoff of 0.05 and the differentially mutated genes are labeled.

(**d**) Ranking 45 genes based on the number of carried hotspot mutations.

(**e-g**) Metastasis-site-specific comparisons of gene copy number variation (CNV) profiles between SU2C and TCGA samples. Each point represents a gene; the x-axis denotes the difference in median CNV values (SU2C – TCGA), and the y-axis shows statistical significance. Dashed vertical lines represent CNV difference thresholds (±0.3), and the horizontal dashed line indicates an adjusted *P*-value cutoff of 0.05.

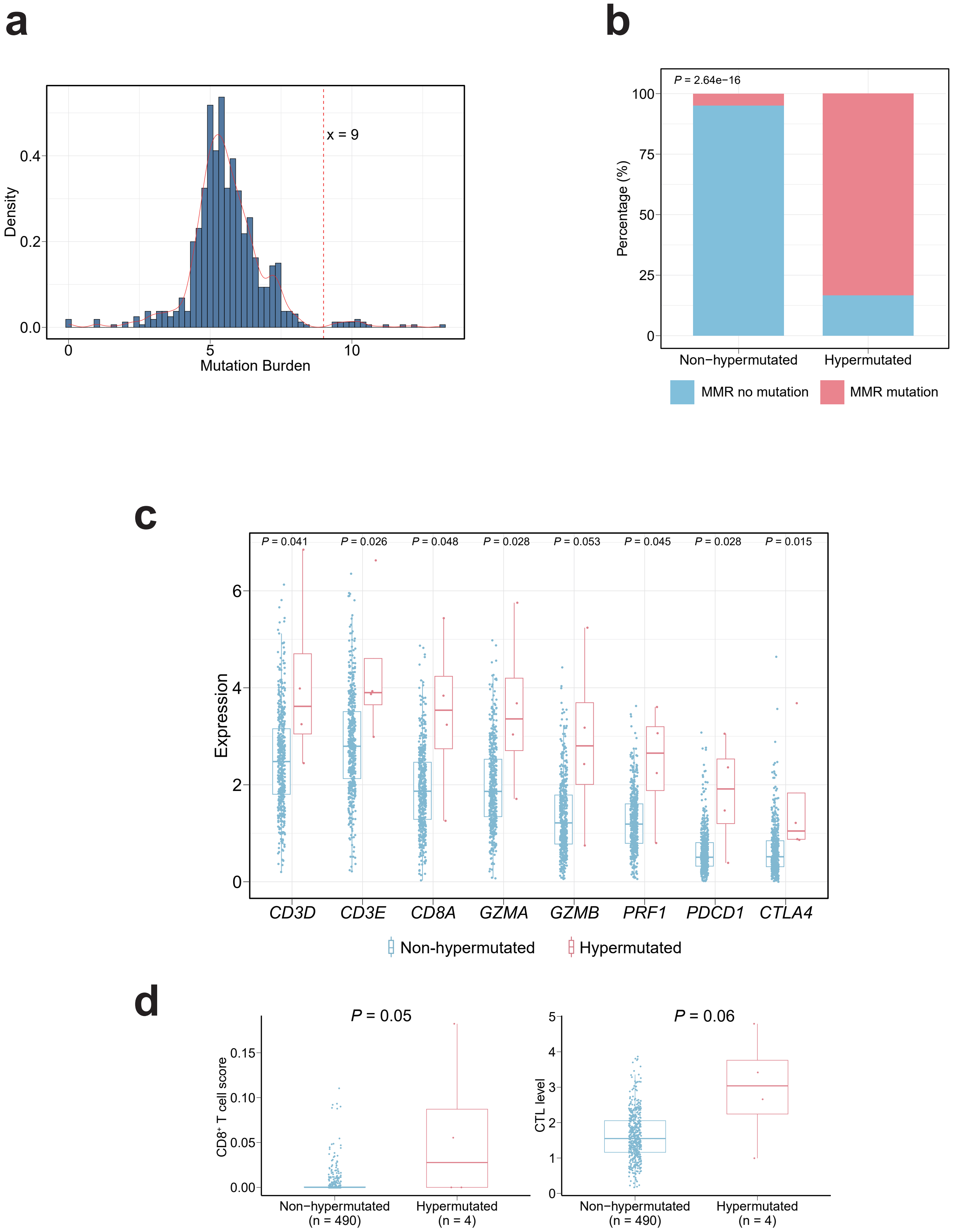
a
Density
0.0
0.2
0.4
0
5
10
Mutation Burden
x = 9
b
P = 2.64e−16
Percentage (%)
100
75
50
25
0
Non−hypermutated
Hypermutated
MMR no mutation
MMR mutation
c
P = 0.041
P = 0.026
P = 0.048
P = 0.028
P = 0.053
P = 0.045
P = 0.028
P = 0.015
Expression
0
2
4
6
CD3D
CD3E
CD8A
GZMA
GZMB
PRF1
PDCD1
CTLA4
Non-hypermutated
Hypermutated
d
P = 0.05
CD8+ T cell score
0.00
0.05
0.10
0.15
Non−hypermutated
(n = 490)
Hypermutated
(n = 4)
P = 0.06
CTL level
0
1
2
3
4
5
Non−hypermutated
(n = 490)
Hypermutated
(n = 4)

Supplementary Figure 2:

(**a**) Histogram of log2-transformed mutation burden values overlaid with a kernel density curve. The dashed vertical line marks the threshold (x = 9) used to define hypermutated samples.

(**b**) Comparison of mutation frequencies of MMR genes between hypermutated and non-hypermutated samples. *P*-value was calculated using Fisher's exact test.

(**c**) Expression of immune-related genes (*CD3D*, *CD3E*, *CD8A*, *GZMA*, *GZMB*, *PRF1*, *PDCD1*, and *CTLA4*) in hypermutated (n=4) and non-hypermutated (n = 490) TCGA samples. In each box, the central line represents the median value and the bounds represent the 25th and 75th percentiles (interquartile range). The whiskers encompass 1.5 times the interquartile range.

(**d**) Comparison of $CD8^+$ T cell scores estimated by the xCell algorithm (left) and cytotoxic T lymphocyte (CTL) levels estimated by the TIDE algorithm (right). In each box, the central line represents the median value and the bounds represent the 25th and 75th percentiles (interquartile range). The whiskers encompass 1.5 times the interquartile range.

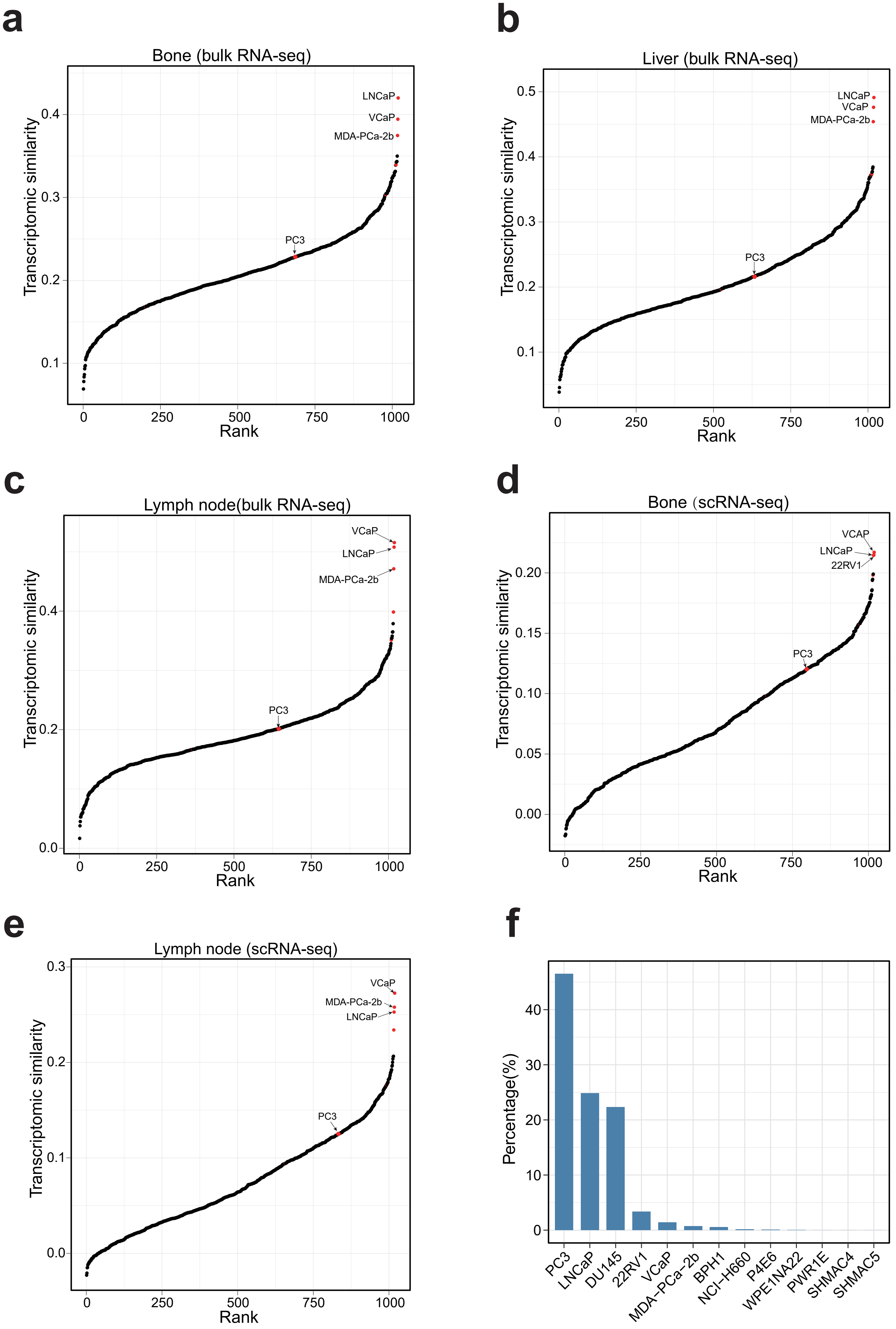
a
Bone (bulk RNA-seq)
Transcriptomic similarity
Rank
LNCaP
VCaP
MDA-PCa-2b
PC3
b
Liver (bulk RNA-seq)
Transcriptomic similarity
Rank
LNCaP
VCaP
MDA-PCa-2b
PC3
c
Lymph node(bulk RNA-seq)
Transcriptomic similarity
Rank
VCaP
LNCaP
MDA-PCa-2b
PC3
d
Bone (scRNA-seq)
Transcriptomic similarity
Rank
VCAP
LNCaP
22RV1
PC3
e
Lymph node (scRNA-seq)
Transcriptomic similarity
Rank
VCaP
MDA-PCa-2b
LNCaP
PC3
f
Percentage(%)
PC3
LNCaP
DU145
22RV1
VCaP
MDA−PCa−2b
BPH1
NCI−H660
P4E6
WPE1NA22
PWR1E
SHMAC4
SHMAC5

Supplementary Figure 3:

(**a-c**) Ranking 1,019 CCLE cell lines based on their transcriptomic similarity to MET500 prostate cancer samples from bone (**a**), liver (**b**), and lymph-node (**c**) metastases. Each dot represents a CCLE cell line, and the prostate cancer cell lines are highlighted in red.

(**d-e**) Ranking 1,019 CCLE cell lines based on their transcriptomic similarity to the malignant cells from bone (**d**) and lymph-node (**e**) metastases in a single-cell RNA-seq dataset. Each dot represents a CCLE cell line, and the prostate cancer cell lines are highlighted in red.

(**f**) Ranking CCLE prostate cancer cell lines based on PubMed citation frequency.

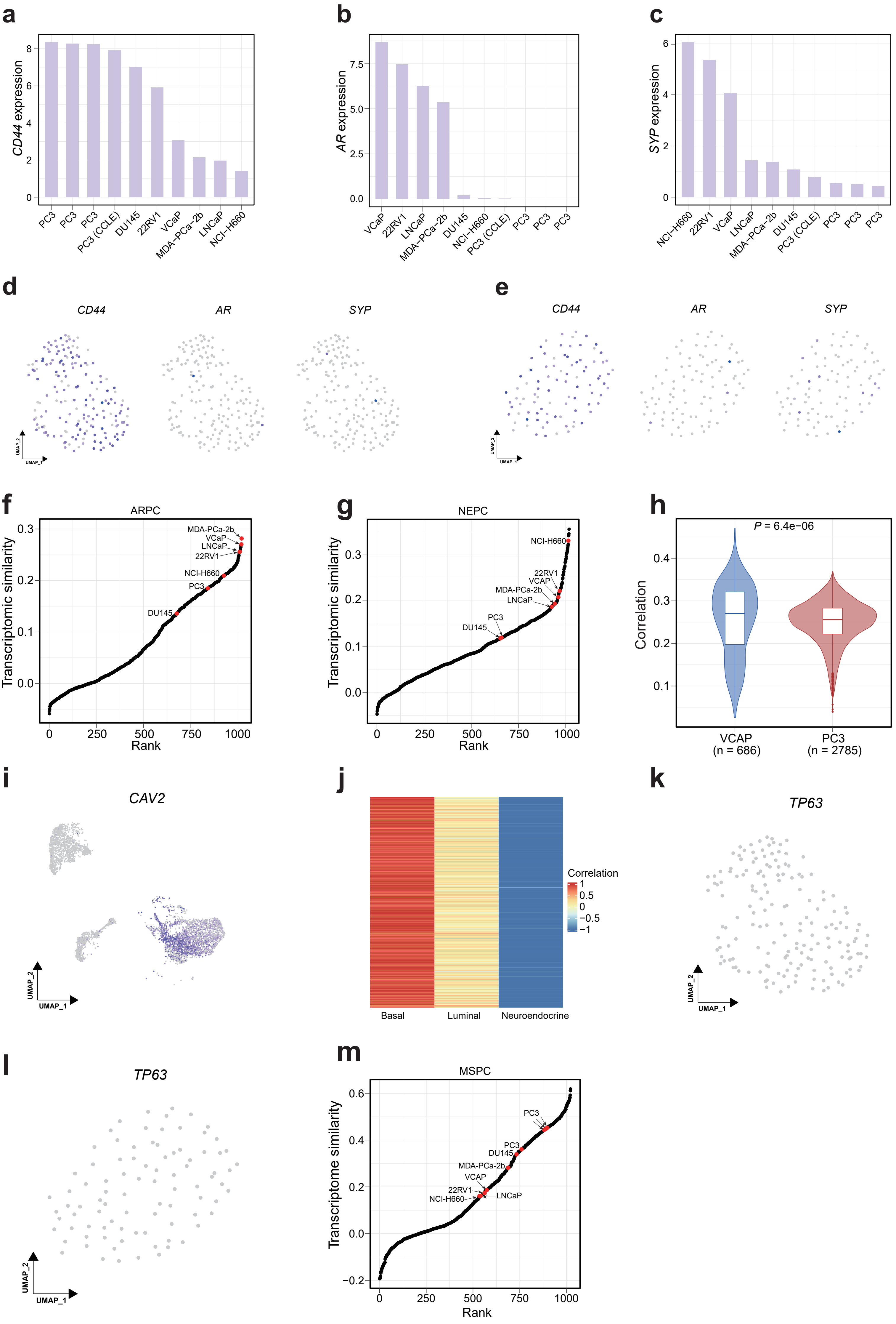

a
CD44 expression
8
6
4
2
0
PC3
PC3
PC3
PC3 (CCLE)
DU145
22RV1
VCaP
MDA-PCa-2b
LNCaP
NCI-H660
b
AR expression
7.5
5.0
2.5
0.0
VCaP
22RV1
LNCaP
MDA-PCa-2b
DU145
NCI-H660
PC3 (CCLE)
PC3
PC3
PC3
c
SYP expression
6
4
2
0
NCI-H660
22RV1
VCaP
LNCaP
MDA-PCa-2b
DU145
PC3 (CCLE)
PC3
PC3
PC3
d
CD44
AR
SYP
UMAP_2
UMAP_1
e
CD44
AR
SYP
UMAP_2
UMAP_1
f
ARPC
Transcriptomic similarity
MDA-PCa-2b
VCaP
LNCaP
22RV1
NCI-H660
PC3
DU145
0.3
0.2
0.1
0.0
0
250
500
750
1000
Rank
g
NEPC
Transcriptomic similarity
NCI-H660
22RV1
VCAP
MDA-PCa-2b
LNCaP
PC3
DU145
0.3
0.2
0.1
0.0
0
250
500
750
1000
Rank
h
P = 6.4e−06
Correlation
0.4
0.3
0.2
0.1
VCAP
(n = 686)
PC3
(n = 2785)
i
CAV2
UMAP_2
UMAP_1
j
Correlation
1
0.5
0
−0.5
−1
Basal
Luminal
Neuroendocrine
k
TP63
UMAP_2
UMAP_1
l
TP63
UMAP_2
UMAP_1
m
MSPC
Transcriptome similarity
PC3
PC3
DU145
MDA-PCa-2b
VCAP
22RV1
NCI-H660
LNCaP
0.6
0.4
0.2
0.0
−0.2
0
250
500
750
1000
Rank

Supplementary Figure 4:

(**a-c**) Expression of key lineage markers *CD44*, *AR* and *SYP* across CCLE prostate cancer cell lines and bulk RNA-seq PC3 samples from dataset GSE116668.

(**d-e**) Expression of *CD44*, *AR*, and *SYP* in PC3 single cells from GSE157220 (**d**) and GSE140440 (**e**). Color represents expression level, from gray (low) to dark blue (high)

(**f-g**) Ranking 1,019 CCLE cell lines based on their transcriptomic similarity to the malignant cells of ARPC (**f**) and NEPC (**g**) subtypes. Each dot represents a CCLE cell line, and the prostate cancer cell lines are highlighted in red.

(**h**) Transcriptomic correlation between VCaP and ARPC malignant cells is significantly higher than that between PC3 and MSPC malignant cells. In each box, the central line represents the median value and the bounds represent the 25th and 75th percentiles (interquartile range). The whiskers encompass 1.5 times the interquartile range. Outliers are shown as individual points.

(**i**) Expression of *CAV2* in malignant cells from the CRPC scRNA-seq dataset. Color represents expression level, from gray (low) to dark blue (high).

(**j**) Heatmap showing the transcriptomic correlation between MSPC malignant cells and three normal prostate epithelial cell types.

(**k-l**) Expression of *TP63* in PC3 single cells from GSE157220 (**k**) and GSE140440 (**l**). All cells show no detectable *TP63* expression (gray).

(**m**) Ranking 1,019 CCLE cell lines and additional PC3 samples (from GSE116668) based on their transcriptomic similarity to the malignant cells of MSPC subtype. Each dot represents a CCLE cell line, and the prostate cancer cell lines are highlighted in red.

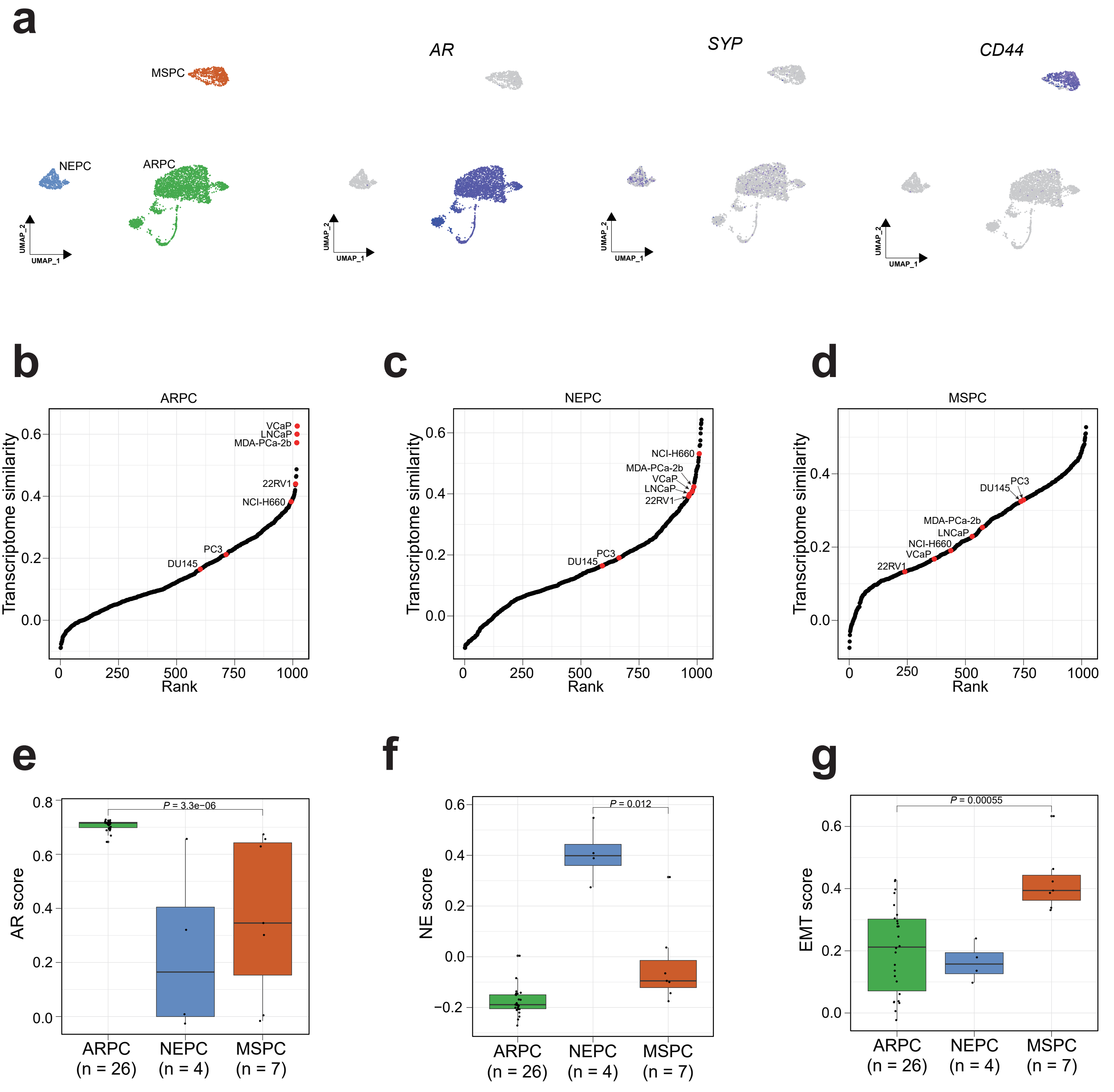
a
MSPC
NEPC
ARPC
UMAP_2
UMAP_1
AR
SYP
CD44
b
ARPC
Transcriptome similarity
VCaP
LNCaP
MDA-PCa-2b
22RV1
NCI-H660
PC3
DU145
Rank
c
NEPC
Transcriptome similarity
NCI-H660
MDA-PCa-2b
VCaP
LNCaP
22RV1
PC3
DU145
Rank
d
MSPC
Transcriptome similarity
PC3
DU145
MDA-PCa-2b
LNCaP
NCI-H660
VCaP
22RV1
Rank
e
AR score
P = 3.3e−06
ARPC (n = 26)
NEPC (n = 4)
MSPC (n = 7)
f
NE score
P = 0.012
ARPC (n = 26)
NEPC (n = 4)
MSPC (n = 7)
g
EMT score
P = 0.00055
ARPC (n = 26)
NEPC (n = 4)
MSPC (n = 7)

Supplementary Figure 5:

(**a**) UMAP visualization of the malignant cells from a scRNA-seq dataset of mCRPC (from GSE210358). The left panel shows subtype annotations and the right three panels depict expression levels of *AR*, *SYP*, and *CD44*, respectively. Color represents expression level, from gray (low) to dark blue (high).

(**b-d**) Ranking 1,019 CCLE cell lines based on their transcriptomic similarity to the malignant cells of ARPC (**b**), NEPC (**c**) and MSPC (**d**) subtypes. Each dot represents a CCLE cell line, and the prostate cancer cell lines are highlighted in red.

(**e-g**) Boxplots to show the *AR* pathway score (**e**), neuroendocrine score (**f**) and EMT score (**g**) for subtyped metastatic prostate cancer samples.

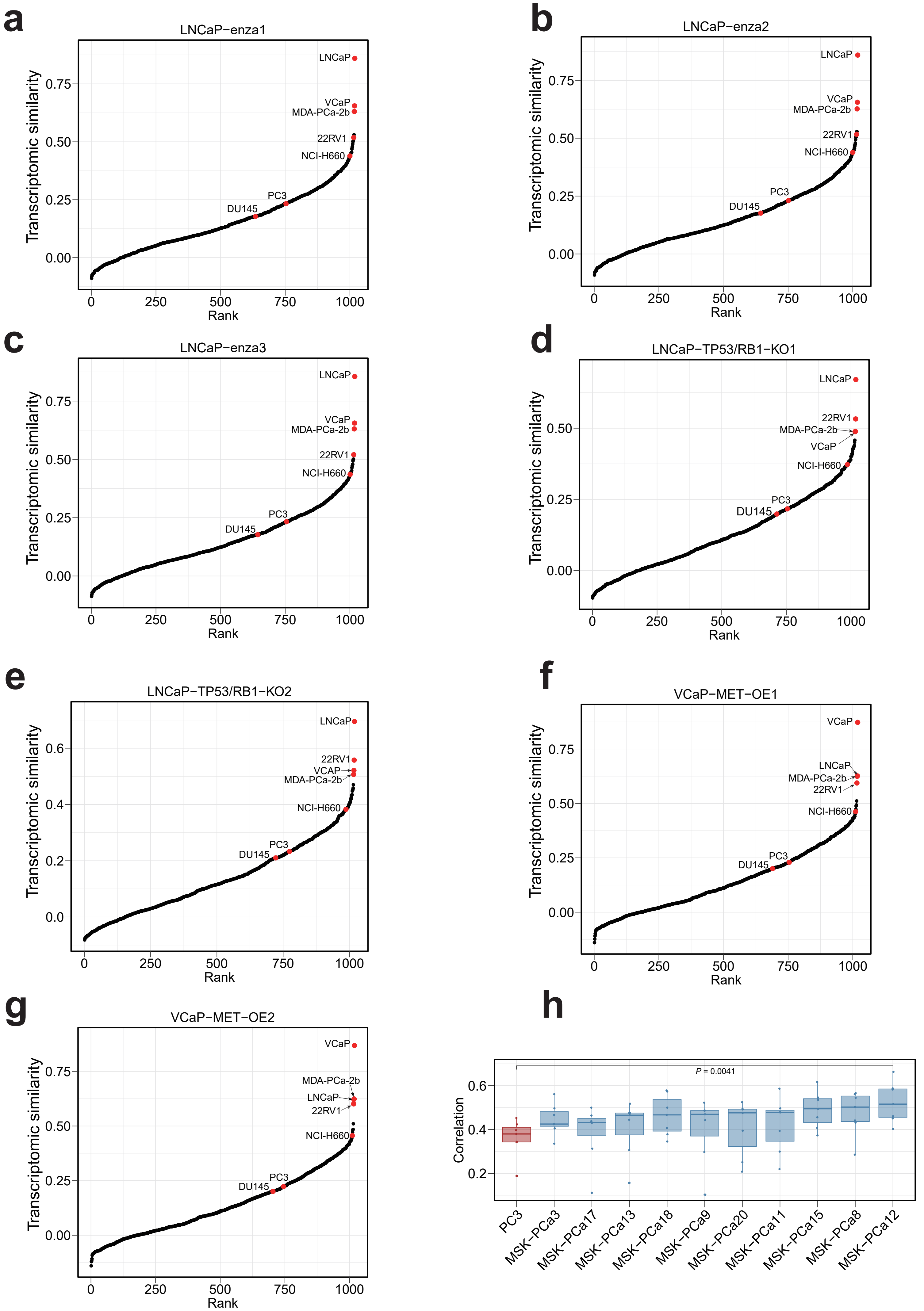

a
LNCaP−enza1
Transcriptomic similarity
Rank
LNCaP
VCaP
MDA-PCa-2b
22RV1
NCI-H660
PC3
DU145
b
LNCaP−enza2
c
LNCaP−enza3
d
LNCaP−TP53/RB1−KO1
e
LNCaP−TP53/RB1−KO2
VCAP
f
VCaP−MET−OE1
g
VCaP−MET−OE2
h
Correlation
P = 0.0041
PC3
MSK−PCa3
MSK−PCa17
MSK−PCa13
MSK−PCa18
MSK−PCa9
MSK−PCa20
MSK−PCa11
MSK−PCa15
MSK−PCa8
MSK−PCa12

Supplementary Figure 6:

(**a-c**) Ranking 1,019 CCLE cell lines based on their transcriptomic similarity to engineered LNCaP cell lines (treated with enzalutamide). Each dot represents a CCLE cell line, and the prostate cancer cell lines are highlighted in red.

(**d-e**) Ranking 1,019 CCLE cell lines based on their transcriptomic similarity to engineered LNCaP cell lines (*TP53*/*RB1* double knockout). Each dot represents a CCLE cell line, and the prostate cancer cell lines are highlighted in red.

(**f-g**) Ranking 1,019 CCLE cell lines based on their transcriptomic similarity to engineered VCaP cell line (*MET* overexpression). Each dot represents a CCLE cell line,

(**h**) Ranking organoids based on their chromatin-accessibility similarity to MSPC patient samples (n = 7). In each box, the central line represents the median value and the bounds represent the 25th and 75th percentiles (interquartile range). The whiskers encompass 1.5 times the interquartile range. Outliers are shown as individual points. *P*-value was calculated using the two-sided Wilcoxon signed-rank test.

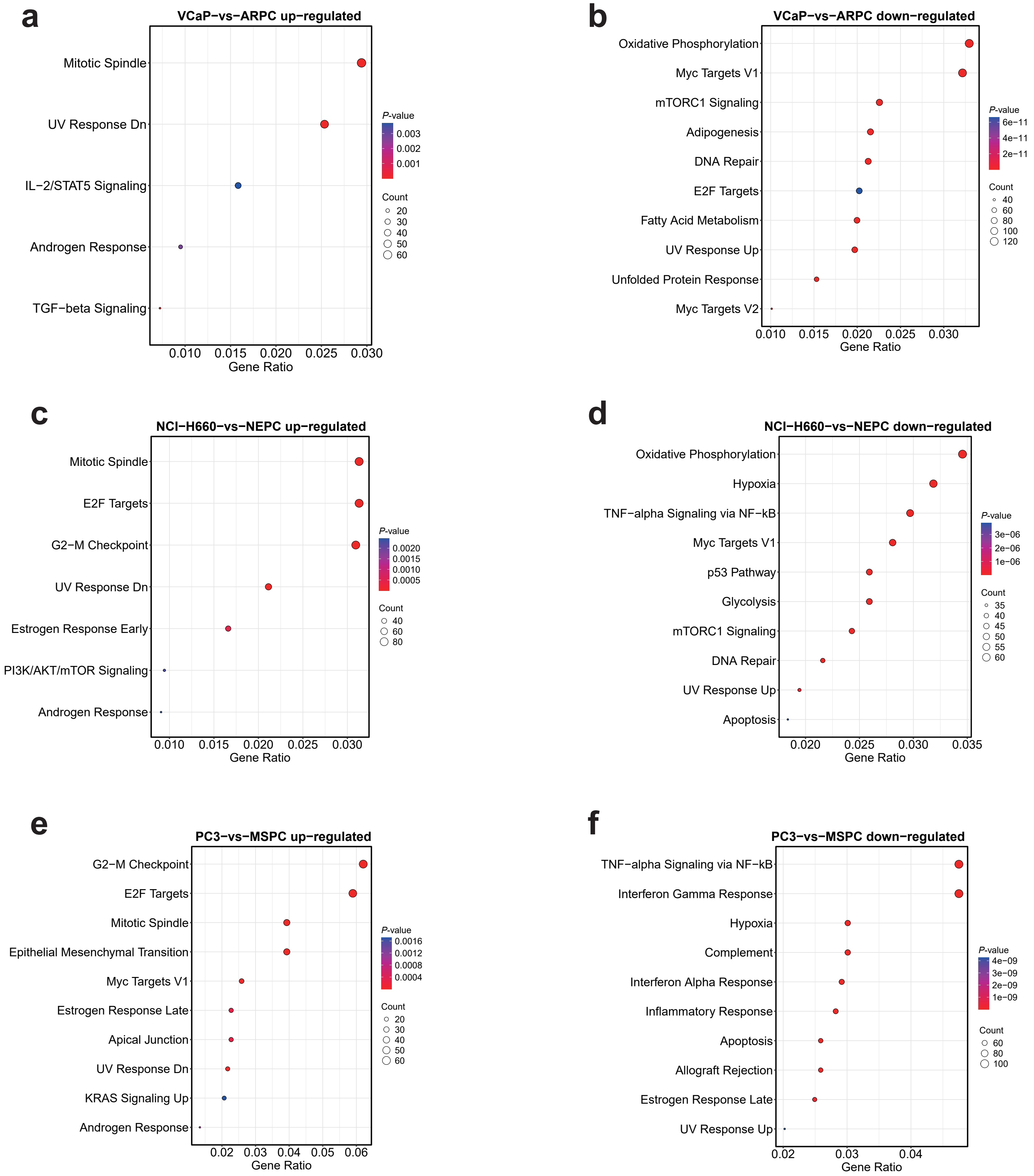
a
VCaP−vs−ARPC up−regulated
Mitotic Spindle
UV Response Dn
IL−2/STAT5 Signaling
Androgen Response
TGF−beta Signaling
0.010
0.015
0.020
0.025
0.030
Gene Ratio
P-value
0.003
0.002
0.001
Count
20
30
40
50
60
b
VCaP−vs−ARPC down−regulated
Oxidative Phosphorylation
Myc Targets V1
mTORC1 Signaling
Adipogenesis
DNA Repair
E2F Targets
Fatty Acid Metabolism
UV Response Up
Unfolded Protein Response
Myc Targets V2
0.010
0.015
0.020
0.025
0.030
Gene Ratio
P-value
6e−11
4e−11
2e−11
Count
40
60
80
100
120
c
NCI−H660−vs−NEPC up−regulated
Mitotic Spindle
E2F Targets
G2−M Checkpoint
UV Response Dn
Estrogen Response Early
PI3K/AKT/mTOR Signaling
Androgen Response
0.010
0.015
0.020
0.025
0.030
Gene Ratio
P-value
0.0020
0.0015
0.0010
0.0005
Count
40
60
80
d
NCI−H660−vs−NEPC down−regulated
Oxidative Phosphorylation
Hypoxia
TNF−alpha Signaling via NF−kB
Myc Targets V1
p53 Pathway
Glycolysis
mTORC1 Signaling
DNA Repair
UV Response Up
Apoptosis
0.020
0.025
0.030
0.035
Gene Ratio
P-value
3e−06
2e−06
1e−06
Count
35
40
45
50
55
60
e
PC3−vs−MSPC up−regulated
G2−M Checkpoint
E2F Targets
Mitotic Spindle
Epithelial Mesenchymal Transition
Myc Targets V1
Estrogen Response Late
Apical Junction
UV Response Dn
KRAS Signaling Up
Androgen Response
0.02
0.03
0.04
0.05
0.06
Gene Ratio
P-value
0.0016
0.0012
0.0008
0.0004
Count
20
30
40
50
60
f
PC3−vs−MSPC down−regulated
TNF−alpha Signaling via NF−kB
Interferon Gamma Response
Hypoxia
Complement
Interferon Alpha Response
Inflammatory Response
Apoptosis
Allograft Rejection
Estrogen Response Late
UV Response Up
0.02
0.03
0.04
Gene Ratio
P-value
4e−09
3e−09
2e−09
1e−09
Count
60
80
100

Supplementary Figure 7:

(**a-f**) MSigDB Hallmark enrichment analysis results for the ARPC (**a-b**), NEPC (**c-d**), and MSPC (**e-f**) subtypes. For each subtype, differentially expressed genes (adjusted *P*-value < 0.05, $|\log_2 FC| > 1$) between the corresponding cell line (VCaP for ARPC, NCI-H660 for NEPC, and PC3 for MSPC) and patient samples were identified using scRNA-seq data. Only significantly enriched terms are shown (adjusted *P*-value < 0.05); when more than ten significant terms were detected, the ten most significant ones are displayed.

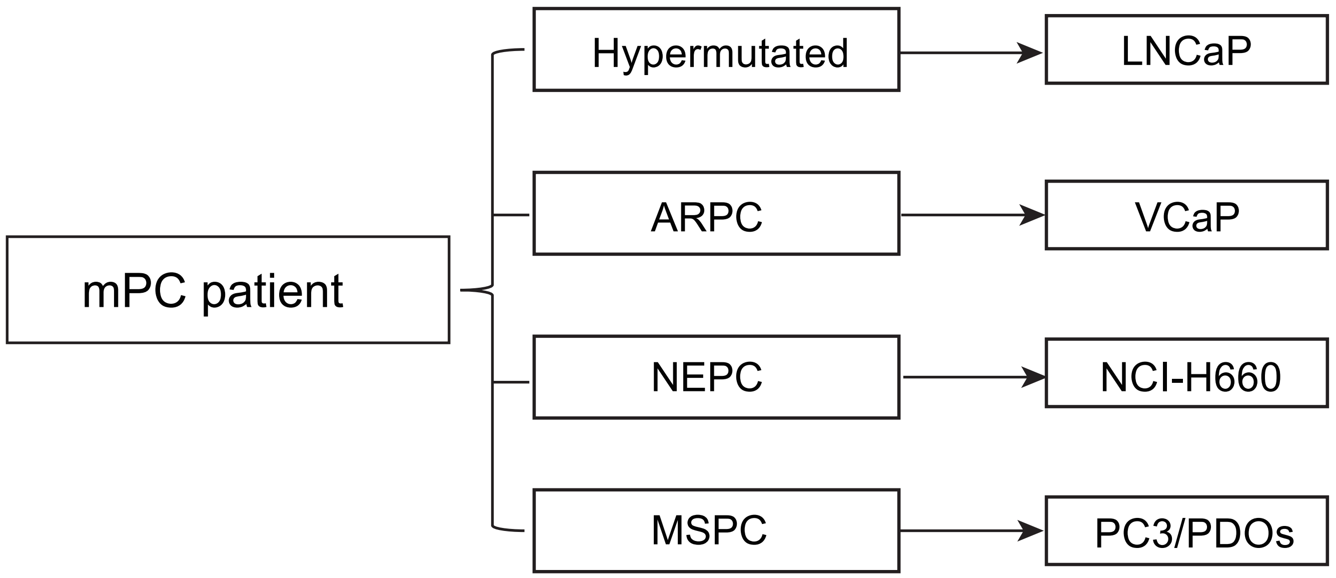
mPC patient
Hypermutated
ARPC
NEPC
MSPC
LNCaP
VCaP
NCI-H660
PC3/PDOs

Supplementary Figure 8:

Overview of suitable prostate cancer cell line models for various metastatic prostate cancer (mPC) research contexts.
