# Supplementary material for: Multi-omics evaluation of cell lines as models for metastatic prostate cancer
Source: Commun Biol. 2026 Mar 24;9:656. doi: 10.1038/s42003-026-09914-2 (PMC13171877; doi:10.1038/s42003-026-09914-2)
Supplement: Supplementary file 13 — Description of Additional Supplementary Files [file 42003_2026_9914_MOESM13_ESM.docx]

**Description of Additional Supplementary File**

File name: Supplementary Data 1

Description: Mutation hotspots.

File name: Supplementary Data 2

Description: Differential gene expression analysis result (hypermutated versus non-hypermutated TCGA samples).

File name: Supplementary Data 3

Description: Hallmark gene sets enrichment of 125 upregulated genes (hypermutated versus non-hypermutated TCGA samples).

File name: Supplementary Data 4

Description: TC analysis result between MET500 prostate cancer samples and 1,019 CCLE cell lines.

File name: Supplementary Data 5

Description: PubMed citation count of CCLE prostate cancer cell lines.

File name: Supplementary Data 6

Description: Hallmark gene sets enrichment of top 200 PC1-loading genes.

File name: Supplementary Data 7

Description: TC analysis result between MSPC malignant cells and 1,019 CCLE cell lines.

File name: Supplementary Data 8

Description: Differential gene expression analysis result (VCAP-MET-OE vs. control).

File name: Supplementary Data 9

Description: Signature genesets.

File name: Supplementary Data 10

Description: Oligonucleotide sequences.

File name: Supplementary Data 11

Description: The source data behind the graphs in the paper.
